# Discovery and Characterization of Antioxidant Compounds in the Pericarp and Seed Byproducts of Different Litchi (*Litchi chinensis* Sonn.) Fruits

**DOI:** 10.3390/antiox15091187

**Published:** 2026-09-17

**Authors:** Yike Huang, Hujie Lyu, Ren-You Gan

**Affiliations:** 1 Department of Food Science and Nutrition, The Hong Kong Polytechnic University, Hung Hom, Hong Kong SAR, China; yikee.huang@connect.polyu.hk (Y.H.); hujie.lyu@connect.polyu.hk (H.L.); 2 Research Centre for Resources Engineering towards Carbon Neutrality (RCRE), The Hong Kong Polytechnic University, Hung Hom, Hong Kong SAR, China; 3 Research Institute for Future Food, The Hong Kong Polytechnic University, Hung Hom, Hong Kong SAR, China

**Keywords:** litchi pericarp, litchi seeds, cultivars, antioxidant activity, total phenolics, procyanidins

## Abstract

The processing of litchi (*Litchi chinensis* Sonn.) fruits generates substantial amounts of pericarp and seed by-products, which are candidate sources of antioxidant compounds but are underutilized. This study systematically investigated the total phenolic content (TPC) and in vitro antioxidant capacities (FRAP, ABTS, and DPPH values) of pericarp and seed extracts across eight commercial litchi cultivars. Qualitative LC-MS-based phytochemical profiling was further performed on the representative ‘Bingli’ cultivar. The results indicated that litchi seeds consistently exhibited significantly higher TPC and antioxidant activities compared to pericarps. Among the tested pericarps, the ‘Nuomici’ cultivar showed the highest TPC (10.29 ± 0.10 mg GAE/g dw) and antioxidant capacities. The ‘Bingli’ cultivar demonstrated the highest overall antioxidant activity among seeds (e.g., TPC of 33.92 ± 1.75 mg GAE/g dw and FRAP of 373.06 ± 4.64 μmol Fe(II)/g dw). LC-MS-based qualitative screening tentatively annotated several hydroxycinnamic acids and A-type procyanidins as putative phenolic constituents of the ‘Bingli’ litchi pericarp and seed extracts. Subsequent targeted HPLC analysis determined the concentrations of Procyanidin A2, revealing that it had a high concentration in the ‘Xianjinfeng’ and ‘Bingli’ seeds (1298.73 ± 54.87 and 1127.81 ± 67.22 μg/g dw, respectively). These findings indicate cultivar- and tissue-specific variations in litchi fruit by-products and suggest that the seeds of ‘Bingli’ and ‘Xianjinfeng’ are candidate sources of antioxidant phenolics for further investigation, pending validation in food models or biological systems.

## 1. Introduction

Litchi (*Litchi chinensis* Sonn.) originates from China and is widely cultivated in tropical and subtropical regions. It has gained considerable consumer acceptance due to its sweet taste and nutrient-rich flesh [1]. However, commercial processing and consumption of litchi fruit generate substantial amounts of agricultural by-products, primarily the pericarp and seeds, which account for approximately 13% to 19% of the fresh fruit’s total weight [2]. These by-products are traditionally discarded as waste, which contributes to environmental pollution and represents a loss of potentially valuable bioresources. As the focus turns toward a circular economy, recent research focuses on upcycling these agro-industrial by-products into high-value, functional ingredients [3].

Beyond their role as agricultural waste, litchi pericarp and seeds are recognized as sources of antioxidant polyphenols, particularly proanthocyanidins [4,5,6]. The pericarp is rich in flavonoids such as epicatechin, procyanidin A2, and procyanidin B2 [7,8]. Similarly, litchi seeds contain procyanidin dimers and trimers [4,9], and other phenolics including gallic acid, (−)-gallocatechin, (−)-epicatechin, and (−)-epicatechin-3-gallate [10]. Several studies have reported strong in vitro antioxidant activity of litchi by-products, in some cases comparable to or higher than conventional benchmarks such as ascorbic acid [8,9]. Furthermore, these by-products have been investigated for other bioactivities, including anticancer, antidiabetic and antimicrobial properties [1,11,12].

Oxidative stress is caused by an overproduction of reactive oxygen species (ROS) and can lead to the deterioration of both biological tissues and food systems. In food products, ROS trigger lipid oxidation, which can generate off-flavors, degrade nutritional quality, and shorten shelf life. In living organisms, oxidative damage to key biomolecules like DNA is linked to chronic diseases, including cancer. To reduce such damage, synthetic antioxidants like butylated hydroxyanisole (BHA) and butylated hydroxytoluene (BHT) have been widely used. However, evidence of their potential health risks, such as carcinogenicity observed in animal models, has turned consumer preference and research interest toward natural and plant-based antioxidant alternatives [13].

Although there are foundational understandings of the phytochemistry of litchi fruit by-products, systematic comparisons of phenolic profiles and antioxidant capacities among the pericarp and seeds across different commercial litchi cultivars are limited. Existing cultivar-comparison studies have mainly focused on litchi pericarp, whereas paired comparisons of pericarp and seeds across multiple commercial cultivars remain scarce. Reported phenolic values may also vary with cultivar, harvest year, maturity, storage, drying, and extraction conditions. Understanding how phenolic phytochemicals vary across cultivars and tissues is therefore important for the targeted application of litchi by-products. This study aimed to determine the TPC and in vitro antioxidant capacities, including ferric reducing antioxidant power (FRAP) and abilities to scavenge 1,1-diphenyl-2-picrylhydrazyl (DPPH) and 2,2′-azinobis (3-ethyl-benzothiazoline-6-sulfonic acid) (ABTS) free radicals, of pericarp and seed extracts across eight litchi cultivars and to compare the differences. Furthermore, non-targeted profiling and targeted quantification were applied to characterize major phenolic compounds in the extracts.

## 2. Materials and Methods

### 2.1. Plant Materials

Eight commercial litchi cultivars (‘Bingli’, ‘Baitangying’, ‘Feizixiao’, ‘Guiwei’, ‘Lizhiwang’, ‘Nuomici’, ‘Qingrenli’, and ‘Xianjinfeng’) were originally produced in Guangdong Province, China, and were purchased from local markets in Qingdao, China. ‘Baitangying’, ‘Feizixiao’ and ‘Lizhiwang’ were purchased in early June 2025, while ‘Bingli’, ‘Guiwei’, ‘Nuomici’, ‘Qingrenli’, and ‘Xianjinfeng’ were purchased in early July 2025 due to their different ripening times as well as availability at the markets. Approximately 2 kg of fresh fruit was collected for each cultivar. The exact number of each variety was not recorded because mature fruits were randomly selected at the market and purchase was standardized by the weight of each variety. After purchase, the fruits were immediately processed within 2 h. The pericarp and seeds were manually separated, cleaned, and stored at −20 °C for further processing.

### 2.2. Chemicals and Reagents

Folin–Ciocalteu reagent was obtained from Sangon Biotech (Shanghai) Co., Ltd. (Shanghai, China). 6-hydroxy-2,5,7,8-tetramethylchroman-2-carboxylic acid (Trolox) was obtained from Sigma-Aldrich (St. Louis, MO, USA). 1,1-diphenyl-2-picrylhydrazyl (DPPH), 2,4,6-Tris(2-pyridyl)-s-triazine (TPTZ), 2,2′-azinobis (3-ethyl-benzothiazoline-6-sulfonic acid) (ABTS), acetonitrile, acetic acid, and ethanol were obtained from Shanghai Aladdin Biochemical Technology Co., Ltd. (Shanghai, China). Gallic acid, Procyanidin B2, and Procyanidin A2 were obtained from Chengdu RefMedic Biotech Co., Ltd. (Chengdu, China). Ultrapure water used was from a Milli-Q water purification system (Merck Millipore, Burlington, MA, USA). All other chemicals used were of analytical grade.

### 2.3. Sample Preparation and Extraction

Samples were dried using a hot-air drying method based on Babbar et al. [14], with temperature selection additionally supported by a recent review on fruit-pomace drying [15], with modifications. Frozen materials were thawed at 4 °C for 2 h, and surface moisture was removed. The pericarp was dried in an oven at 60 °C for 6 h, and the seeds were dried at 60 °C for 10 h. Drying was continued until constant weight was reached. The dried tissues were milled into fine powders and stored at 4 °C.

Phenolic extraction [16,17,18] was performed according to the method described by Soni et al. [19] with minor modifications. Briefly, 0.5 g of dry sample powder was mixed with 10 mL of 70% ethanol (1:20, *w*/*v*) in a 15 mL centrifuge tube. Extraction was carried out in a dry shaker at 37 °C for 24 h. All extractions were performed in triplicate. After extraction, the mixtures were centrifuged at 3400× *g* for 20 min, and the supernatants were collected for analysis.

### 2.4. Determination of TPC

The TPC of the extracts was estimated using the Folin–Ciocalteu method [20] adopted from Li et al. [21] with minor modifications. Briefly, the extracts were diluted to an appropriate concentration, and 100 μL of the sample was transferred into 2 mL centrifuge tubes. Subsequently, 400 μL of deionized water and 100 μL of Folin–Ciocalteu reagent were sequentially added and mixed thoroughly via vortexing. After 5 min of incubation in the dark at room temperature, 1000 μL of 7% (*w*/*v*) sodium carbonate solution was added. The mixture was vortexed and incubated in the dark at room temperature for 90 min. After incubation, absorbance was measured at 760 nm using a microplate reader. Gallic acid (20–140 μg/mL) was used to construct the standard curve. The calibration curve was linear over this range, with the regression equation y = 0.0056x + 0.0366 (R^2^ = 0.9993). All sample absorbances were within the linear range of the calibration curve. The TPC was expressed as milligrams of gallic acid equivalents per gram of sample dry weight (mg GAE/g dw).

### 2.5. Determination of Antioxidant Capability

#### 2.5.1. Ferric Reducing Antioxidant Power (FRAP) Assay

The FRAP assay [22] was performed based on the method described by Liu et al. [23] with minor modifications. The working FRAP solution was freshly prepared by mixing 20 mmol/L FeCl_3_·6H_2_O solution, 10 mmol/L TPTZ solution (in 40 mM HCl), and 300 mmol/L sodium acetate buffer (pH 3.6) in a 1:1:10 (*v*/*v*/*v*) ratio and warmed to 37 °C before use. After the preparation, 40 μL of appropriately diluted extract was mixed with 1200 μL of the FRAP working solution. The mixture was vortexed and incubated in the dark at room temperature for 4 min. Absorbance was recorded at 593 nm using a microplate reader. Ferrous sulfate (200–1000 μmol/L) served as the standard, and the results were expressed as μmol of ferrous sulfate equivalents per gram of dry weight (μmol Fe(II)/g dw).

#### 2.5.2. ABTS Radical Scavenging Capability Assay

The ABTS radical scavenging capacity [24] was evaluated according to Mai et al. [25] with minor modifications. The ABTS radical cation (ABTS^+^) was generated by reacting 7 mmol/L ABTS solution with 2.45 mmol/L potassium persulfate (K_2_S_2_O_8_) in a 1:1 (*v*/*v*) ratio. The mixture was incubated in the dark at room temperature for 12–16 h. Before use, the stock solution was diluted with deionized water to achieve an absorbance of 0.70 ± 0.05 at 734 nm. After dilution, 40 μL of diluted sample extract was mixed with 1560 μL of the ABTS working solution. The mixture was incubated in the dark for 6 min, and the absorbance was measured at 734 nm. Trolox (50–1000 μmol/L) was used as the standard, and the results were expressed as μmol Trolox equivalents per gram of dry weight (μmol TE/g dw).

#### 2.5.3. DPPH Radical Scavenging Capability Assay

The DPPH radical scavenging activity [26] was determined using a modified protocol from Yang et al. [27]. A stock solution of 0.1 mmol/L DPPH was prepared and diluted to obtain an absorbance of 0.70 ± 0.05 at 515 nm. In total, 40 μL of diluted extract was added to 1560 μL of the DPPH working solution. After vortexing, the mixture was incubated in the dark at room temperature for 2 h. The absorbance was then measured at 515 nm. Trolox (50–1000 μmol/L) was used as the standard, and the radical scavenging capacity was expressed as μmol TE/g dw.

### 2.6. Identification of Antioxidant Polyphenols by UHPLC-Q-TOF/MS

Ultra-high-performance liquid chromatography quadrupole time-of-flight mass spectrometry (UHPLC-Q-TOF/MS) was performed using an Agilent 6540 system (Agilent Technologies, Santa Clara, CA, USA). Chromatographic separation was achieved on a Waters ACQUITY UPLC BEH C18 column (2.1 mm × 50 mm, 1.7 µm) maintained at 50 °C. The mobile phase consisted of solvent A (water/acetonitrile, 60:40, *v*/*v*, containing 0.1% formic acid) and solvent B (acetonitrile/isopropanol, 50:50, *v*/*v*, containing 0.1% formic acid). The gradient elution program was set as follows: 20% B (0–1 min), 20–99% B (1–8 min), 99% B (8–10 min), 99–20% B (10–10.1 min), and 20% B (10.1–13 min). The flow rate was 0.3 mL/min, and the injection volume was 3 μL. Mass spectrometry was operated in the negative electrospray ionization (ESI^−^) mode. Full-scan mass spectra were acquired over a mass-to-charge (*m*/*z*) range of 50–1700. Data acquisition and processing were conducted using Agilent MassHunter Workstation software version B.06.00. Raw data were subsequently imported into Progenesis QI software version 3.1.9211.37750 for analysis, and compound identification was performed by comparing precursor and fragment ions with the METLIN™ MS/MS Library (2019), the ChemSpider database, the HMDB library, and corroborating with existing literature.

### 2.7. Quantification of Main Phenolic Compounds by HPLC

High-performance liquid chromatography (HPLC) analysis was performed on an Agilent 1260 Infinity II system equipped with a diode-array detector (DAD) (Agilent Technologies, Santa Clara, CA, USA). Separation was carried out on an Agilent ZORBAX SB-C18 column (4.6 mm × 150 mm, 5 µm). The method was adapted from Li et al. [21]. The mobile phase consisted of a 0.4% (*v*/*v*) aqueous acetic acid solution (solvent A) and pure acetonitrile (solvent B). The gradient elution profile was programmed as follows: 5–15% B (0–20 min), 15–25% B (20–40 min), 25–35% B (40–45 min), 35–50% B (45–50 min), and 50–5% B (50–55 min). The injection volume was 10 μL, and the flow rate was maintained at 1 mL/min. Flavan-3-ols were detected at a wavelength of 280 nm. Procyanidin B2 (12.5, 25, and 50 μg/mL) and Procyanidin A2 (5, 12.5, 25, and 50 μg/mL) were used as external standards for quantification. The content of each identified phenolic compound was expressed as μg/g dry weight (μg/g dw).

### 2.8. Statistical Analysis

All the experiments were conducted in triplicate and the data were calculated and analyzed by GraphPad Prism version 10.2.1 (GraphPad Software, Boston, MA, USA), and were mainly expressed as the mean ± standard deviation. One-way analysis of variance (ANOVA) plus the *post hoc* Tukey’s honestly significant difference (HSD) test was used for multiple comparisons among the means. Differences were considered statistically significant at *p* < 0.05.

## 3. Results

### 3.1. TPC of the Pericarp and Seeds

**Figure 1:**
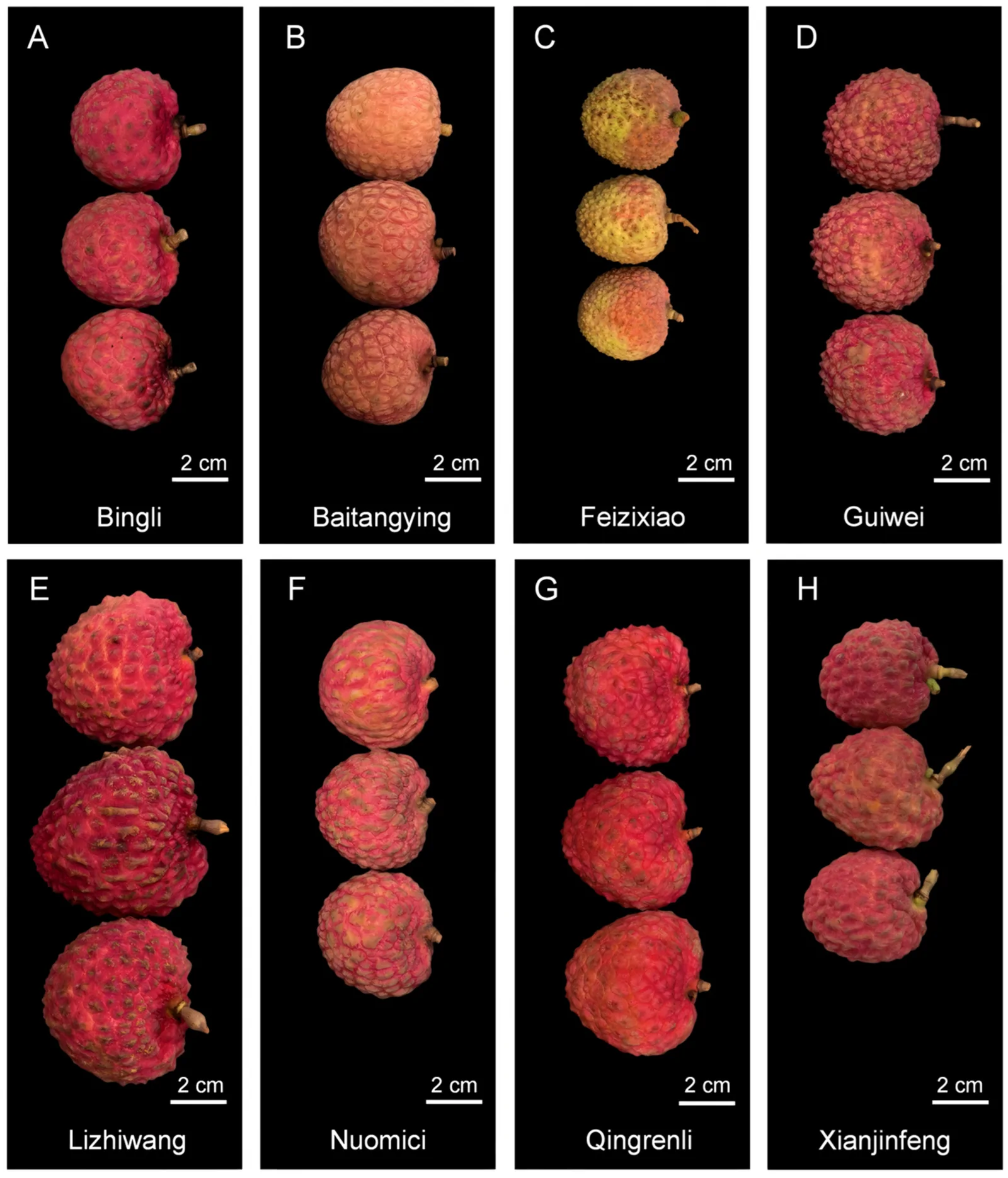
Representative photographs of fresh litchi fruits from eight cultivars. (**A**) Bingli; (**B**) Baitangying; (**C**) Feizixiao; (**D**) Guiwei; (**E**) Lizhiwang; (**F**) Nuomici; (**G**) Qingrenli; (**H**) Xianjinfeng. Scale bar = 2 cm.

Representative photographs of the eight litchi cultivars used in this study are shown in Figure 1. The fruits showed visible cultivar-dependent differences in external appearance, including fruit size, shape, pericarp color, and surface texture. These visual differences provide a morphological reference for the subsequent comparison of phenolic contents, antioxidant capacities, and phytochemical profiles among cultivars.

**Figure 2:**
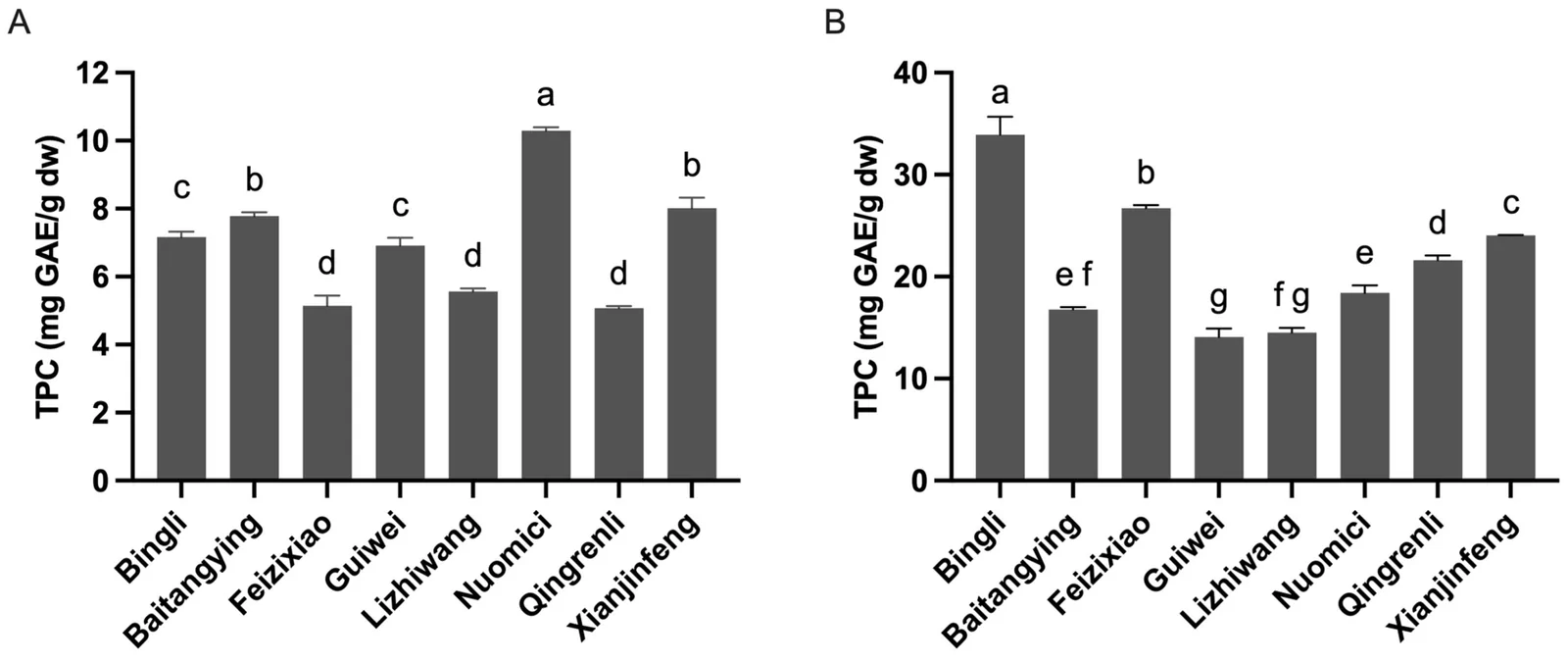
TPC in litchi by-products of eight cultivars (mean ± standard deviation, n = 3). (**A**) TPC in pericarp; (**B**) TPC in seeds. Different letters on the top of columns indicate significant difference at *p* < 0.05. GAE: gallic acid equivalents.

Significant variations in TPC were observed across the eight litchi cultivars for both pericarp and seeds (Figure 2A,B). Seed extracts exhibited higher TPC (14.08–33.92 mg GAE/g dw) compared to pericarp extracts (5.07–10.29 mg GAE/g dw). Among the pericarps, the ‘Nuomici’ cultivar exhibited the highest TPC (10.29 ± 0.10 mg GAE/g dw) and ‘Qingrenli’ showed the lowest. In the seeds, the ‘Bingli’ cultivar demonstrated the highest TPC (33.92 ± 1.75 mg GAE/g dw) and the ‘Guiwei’ cultivar had the lowest across all tested samples.

### 3.2. Antioxidant Capability

**Figure 3:**
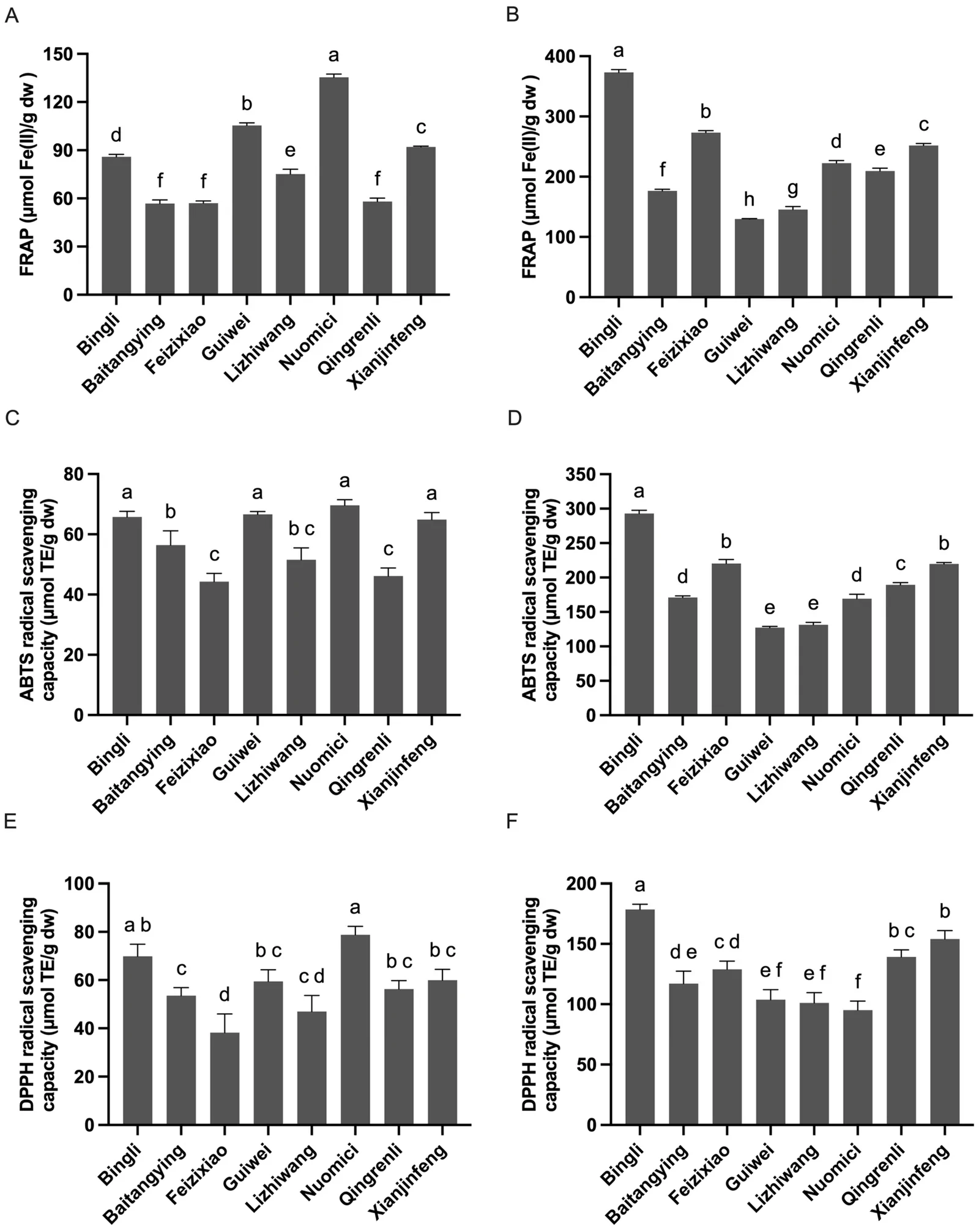
Antioxidant capacities of litchi by-products from eight cultivars (mean ± standard deviation, n = 3). (**A**) FRAP of pericarps; (**B**) FRAP of seeds; (**C**) ABTS of pericarps; (**D**) ABTS of seeds; (**E**) DPPH of pericarps; (**F**) DPPH of seeds. Different letters on the top of columns indicate significant difference at *p* < 0.05. Fe(II): ferrous sulfate equivalents; TE: Trolox equivalents.

The in vitro antioxidant capacities of the litchi by-product extracts exhibited significant variations among cultivars (Figure 3A–F), which were evaluated via FRAP, ABTS, and DPPH assays. Furthermore, consistent with the TPC results, litchi seeds demonstrated significantly higher antioxidant capacity than pericarps across all three assays.

In the FRAP assay (Figure 3A,B), pericarps showed a reducing power ranging from 56.82 to 135.39 μmol Fe(II)/g dw, and the ‘Nuomici’ cultivar showed the highest values (135.39 ± 2.09 μmol Fe(II)/g dw). Seeds showed higher reducing power than pericarps (130.15–373.06 μmol Fe(II)/g dw), where the ‘Bingli’ cultivar seed exhibited the highest value (373.06 ± 4.64 μmol Fe(II)/g dw) compared to other seed samples.

For the ABTS radical scavenging assay (Figure 3C,D), pericarp values ranged from 44.32 to 69.61 μmol TE/g dw, with the ‘Bingli’, ‘Guiwei’, ‘Nuomici’, and ‘Xianjinfeng’ cultivars showing comparably high activities. Seed values ranged from 126.88 to 292.93 μmol TE/g dw, with the ‘Bingli’ cultivar again showing the highest scavenging capacity (292.93 ± 4.82 μmol TE/g dw).

Similarly, the DPPH assay (Figure 3E,F) identified the ‘Nuomici’ and ‘Bingli’ cultivar pericarps (78.80 ± 3.53 μmol TE/g dw and 69.85 ± 5.00 μmol TE/g dw, respectively) and the ‘Bingli’ cultivar seed (178.57 ± 4.32 μmol TE/g dw) as having the highest scavenging activities in their respective groups. Considering the combined results of TPC and the three antioxidant assays, the ‘Bingli’ cultivar was selected for subsequent phytochemical identification and targeted quantification due to its highest overall antioxidant activity, particularly in the seed fraction.

**Figure 4:**
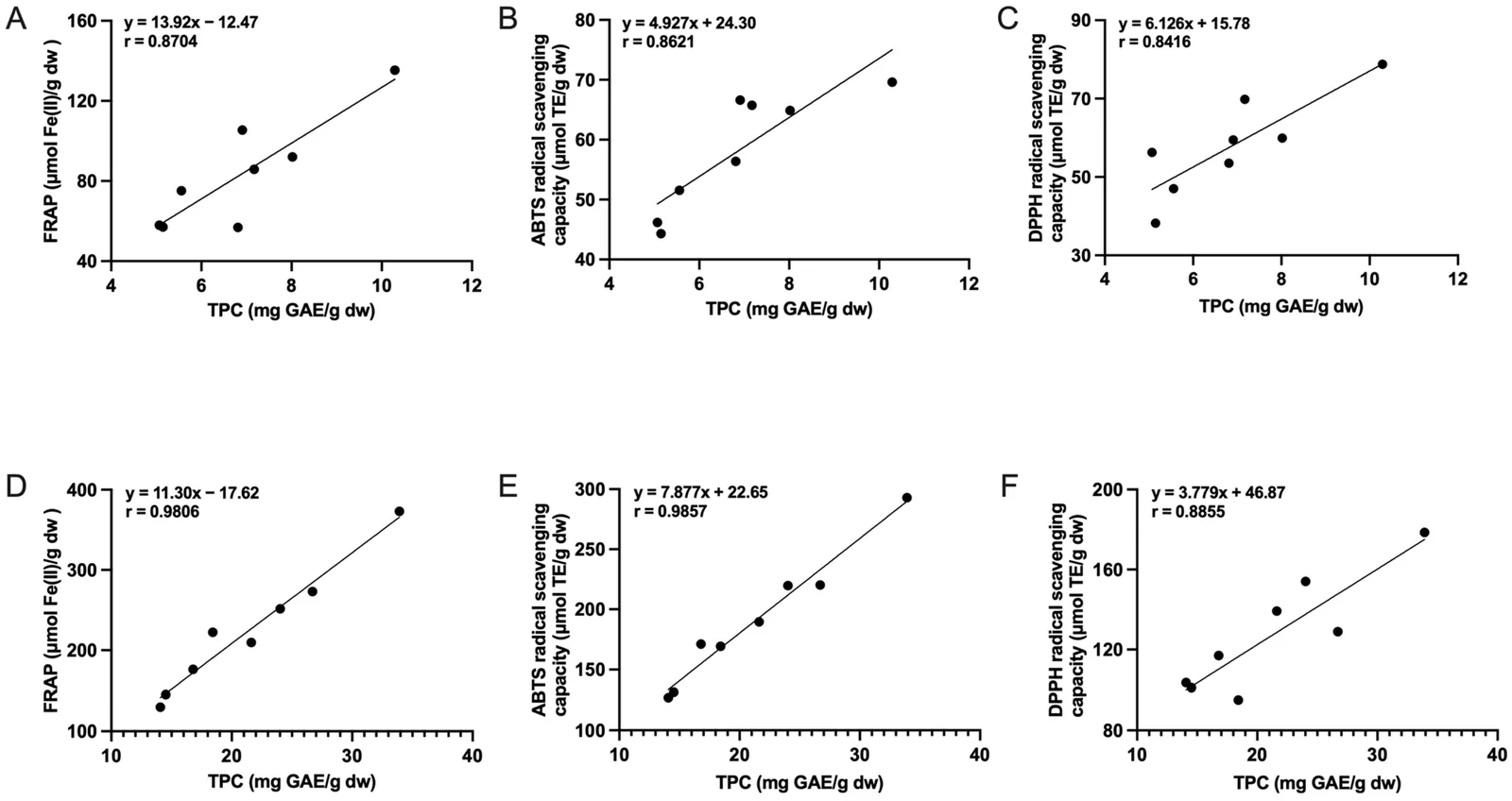
Correlations between TPC and antioxidant capacity assays of litchi by-products from eight cultivars. (**A**) Correlation between TPC and FRAP in pericarp samples (r = 0.8704, *p* = 0.0049, 95% CI: 0.43–0.98); (**B**) correlation between TPC and ABTS in pericarp samples (r = 0.8621, *p* = 0.0059, 95% CI: 0.40–0.97); (**C**) correlation between TPC and DPPH in pericarp samples (r = 0.8416, *p* = 0.0088, 95% CI: 0.34–0.97); (**D**) correlation between TPC and FRAP in seed samples (r = 0.9806, *p* < 0.0001, 95% CI: 0.89–0.997); (**E**) correlation between TPC and ABTS in seed samples (r = 0.9857, *p* < 0.0001, 95% CI: 0.92–0.998); (**F**) correlation between TPC and DPPH in seed samples (r = 0.8855, *p* = 0.0034, 95% CI: 0.48–0.98). Because the sample size was small (n = 8), the confidence intervals were relatively wide, especially for pericarp correlations, and the results should be interpreted with caution.

Furthermore, the results of correlation analysis between TPC and antioxidant assays (FRAP, ABTS, and DPPH) all showed positive correlations (Figure 4A–F). In the pericarp, their r values were 0.8704, 0.8621, and 0.8416 (Figure 4A–C), respectively, and their r values were 0.9806, 0.9857, and 0.8855 (Figure 4D–F), respectively, in the seeds. Because the analysis was based on eight cultivar-level observations, these correlations should be interpreted as associations rather than evidence of causality.

### 3.3. Qualitative and Quantitative Analyses of Antioxidant Polyphenols in Litchi Pericarp and Seeds

LC-MS-based qualitative screening of ‘Bingli’ cultivar extracts suggested the tentative presence of several phenolic compounds in both pericarp and seed tissues (Table 1), including different coumaroylquinic acid isomers, procyanidins, cinnamtannins, and pavetannins. Compound annotation was based on comparison of precursor and fragment ions with METLIN, ChemSpider, and HMDB databases and with literature data. Since authentic standards were not available for most of these compounds, the identifications are tentative, especially for isomers. The complete lists of tentatively identified compounds of litchi by-products are shown in Table S1. Although many compounds were tentatively identified, previous studies have established that A-type proanthocyanidin dimers, particularly Procyanidin A2, are the principal bioactive oligomers in litchi tissues [4,21]. Therefore, targeted HPLC quantification was focused on dimeric structures to obtain the main phytochemical characteristics of the extracts. Procyanidin A2 was selected as the principal analytical biomarker for A-type linkages, and Procyanidin B2 was also checked since it was also reported in some previous studies [10,21,28].

**Figure 5:**
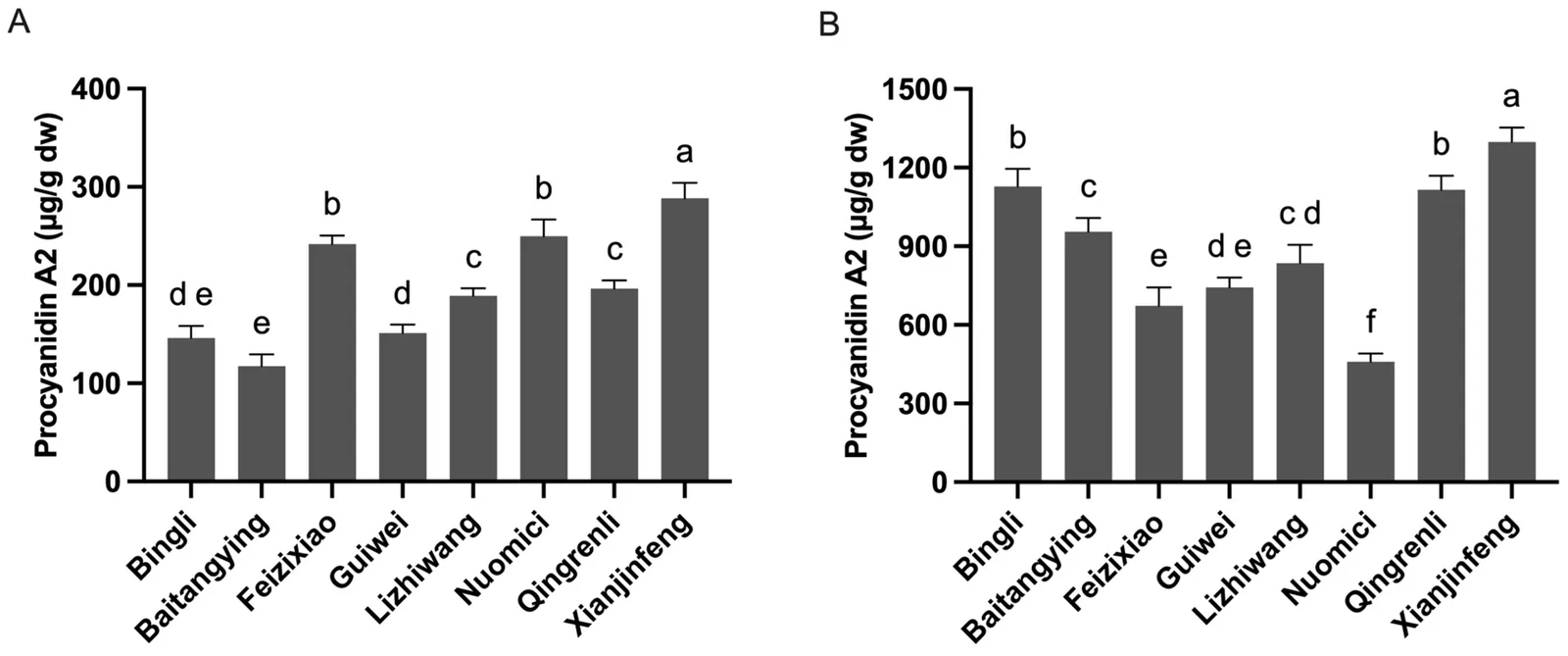
Procyanidin A2 in litchi by-products of eight cultivars (mean ± standard deviation, n = 3). (**A**) Procyanidin A2 in pericarps; (**B**) Procyanidin A2 in seeds. Different letters on the top of columns indicate significant difference at *p* < 0.05.

**Figure 6:**
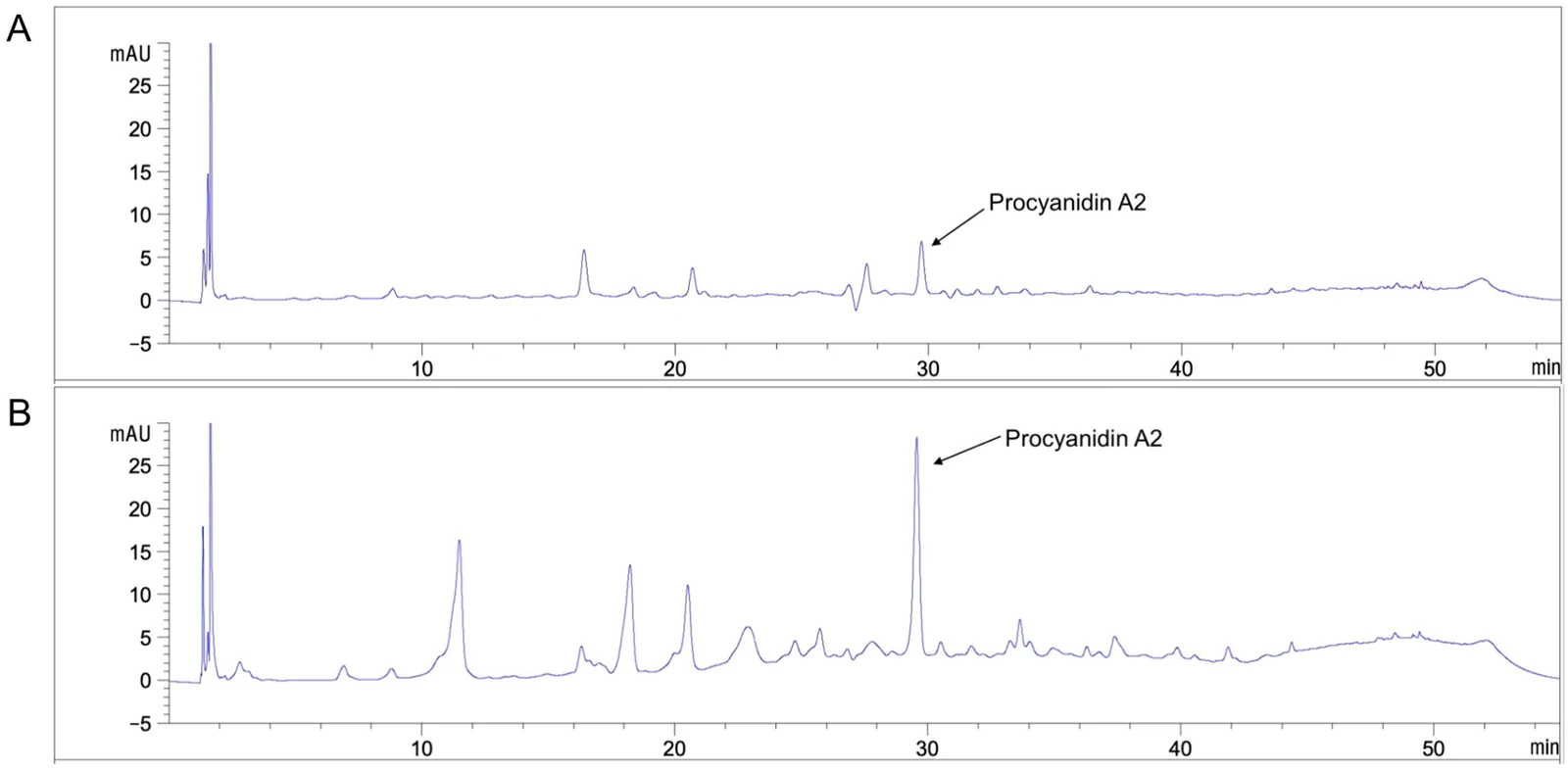
HPLC profile of extracts from the ‘Xianjinfeng’ cultivar. (**A**) HPLC profile of pericarp. (**B**) HPLC profile of seeds. Retention time (*t*_R_) of Procyanidin A2 is about 29 min.

As shown in Figure 5, Procyanidin A2 was detected in all samples with significant inter-cultivar variations, and its standard calibration curve equation is y = 6.3818x − 2.6569 (R^2^ = 0.99995). Litchi seeds exhibited a higher Procyanidin A2 concentration (459.93–1298.73 μg/g dw) compared to the pericarps (117.57–288.52 μg/g dw). The highest value was observed in the ‘Xianjinfeng’ cultivar, which was 1298.73 ± 54.87 μg/g dw in the seeds and 288.52 ± 15.53 μg/g dw in the pericarp. The lowest concentration of Procyanidin A2 in pericarp was in ‘Baitangying’ (117.57 ± 11.79 μg/g dw) and in seeds was in ‘Nuomici’ (459.93 ± 30.13 μg/g dw). The HPLC profile of ‘Xianjinfeng’ is shown in Figure 6. The calibration curve equation of Procyanidin B2 standard is y = 5.0289x − 17.718 (R^2^ = 0.99980); however, it was not detected in any of the samples analyzed under the current conditions. Since the purpose of this analysis was simply to quantify Procyanidin A2 and B2 by external calibration, the calibration curve was highly linear (R^2^ = 0.99995 and R^2^ = 0.99980, respectively), and no recovery issue was involved, so we did not perform additional validation for LOD, LOQ, recovery, or precision.

## 4. Discussion

### 4.1. Analysis of TPC

The TPC estimated by the Folin–Ciocalteu method exhibited significant cultivar- and tissue-dependent variations. Across all eight cultivars, litchi seeds showed higher measured TPC than the pericarps under the present drying and extraction conditions. Among the test samples, ‘Nuomici’ exhibited the highest phenolic contents in the pericarp, and ‘Bingli’ showed the highest in the seeds. These inter-cultivar variations are consistent with previous studies. For instance, Wang et al. [29] reported that the TPC in the pericarps of ten cultivars ranged from 51.3 to 102.1 mg GAE/g dw across different harvest years, and Li et al. [21] observed values between 9.39 and 30.16 mg GAE/g fw among nine cultivars, and both studies showed significant cultivar-dependent variation. The numerical differences between the present study and previous studies can be primarily attributed to pre-harvest biological variables (e.g., climatic conditions and harvest maturity) and post-harvest methodological differences, such as storage conditions, drying techniques, and extraction protocols.

Furthermore, the Folin–Ciocalteu assay is not strictly specific for phenolic compounds and may react with other reducing substances present in the extracts. Therefore, it essentially measures the overall reducing capacity rather than the absolute polyphenol content [30]. Although there are numerical differences, the high ranking of ‘Nuomici’ pericarp TPC in both the present study and Wang et al. [29] is consistent with previous observations. This agreement should be interpreted as trend consistency rather than independent validation of the present results. Cross-study TPC values are used here only to provide context, but not direct comparison, since differences in sample preparation, moisture basis, extraction, and analytical procedures prevent direct ranking across studies.

Besides inter-cultivar variations, the TPC values in litchi pericarp and seeds were considered alongside published values for other agricultural by-products. Reported TPC values include 3.31 to 23.46 mg GAE/g dw for citrus peels [31], 27.7 ± 3.4 mg GAE/g dw for jackfruit seed, and 3.68 ± 0.05 mg GAE/g dw for kinnow seed [14,32]. However, differences in cultivar, sample preparation, moisture basis, extraction conditions, and analytical procedures preclude direct ranking across studies. These values are therefore presented only as contextual benchmarks and do not demonstrate that litchi by-products have higher or superior phenolic content.

### 4.2. Analysis of Antioxidant Capacity

The in vitro antioxidant capacities of the extracts varied with their phenolic profiles, although assay-dependent differences were observed among the cultivars. ‘Nuomici’ pericarp consistently demonstrated the highest antioxidant activities in the FRAP and DPPH assays, which is similar to the findings of Wang et al. [29]. The ABTS assay showed comparably high pericarp activities across ‘Bingli’, ‘Guiwei’, ‘Nuomici’, and ‘Xianjinfeng’, indicating that the choice of assay affected pericarp ranking more strongly than seed ranking. In contrast, ‘Bingli’ seeds showed consistently high activity across the FRAP, ABTS, and DPPH assays.

Such intra-assay variations are commonly observed due to the different reaction mechanisms of antioxidant assays. The FRAP assay evaluates reducing potential via single-electron transfer, which generally correlates well with total antioxidant capacity in litchi tissues [29]. In contrast, the discrepancies between the radical scavenging DPPH and ABTS assays can be attributed to molecular kinetics and solvent interactions. DPPH free radicals exhibit significant steric hindrance, meaning bulkier polyphenolic molecules may react slowly or incompletely with the radical [33]. Additionally, the ABTS assay captures both hydrophilic and lipophilic antioxidant capacities, whereas the DPPH assay predominantly reflects hydrophobic interactions [33]. Consequently, this multi-assay approach provides a more comprehensive evaluation of the in vitro antioxidant potential of the extracts.

Published antioxidant-capacity values for other fruit residues were considered only as contextual benchmarks. Reported values include 25.07 to 461.72 μmol TE/g dw in the ABTS assay and 21.80 to 357.18 μmol TE/g dw in the DPPH assay for citrus peels [31], 5.67 ± 0.32 mg TE/g dw in the ABTS assay for banana peels [14], and 2.8 ± 0.3 μmol Fe(II)/g dw in the FRAP assay for jackfruit seeds [32]. Differences in sample matrix, extraction procedure, moisture basis, units, and assay conditions preclude direct comparison or ranking across these studies. Accordingly, the cited values do not support a conclusion that litchi by-products have better antioxidant activity. Overall, the positive correlations between the antioxidant assay results and TPC suggest an association between Folin–Ciocalteu-estimated reducing capacity and in vitro antioxidant responses. Because TPC is a summary parameter and correlation does not establish causality, these results should not be interpreted as proof that phenolic compounds alone caused the observed antioxidant activity. Non-phenolic reducing substances and synergistic effects among specific flavonoids may also have contributions [14].

### 4.3. Qualitative and Quantitative Analysis of Litchi Extracts

The ‘Bingli’ cultivar was selected for qualitative profiling based on its combined performance across both tissues. Its seeds ranked first in all antioxidant assays, and its pericarp also showed high activity in TPC and antioxidant assays. In contrast, other cultivars with high pericarp activity, such as ‘Nuomici’, showed considerably lower seed performance. Since the present study aimed to evaluate both pericarp and seeds as potential sources of antioxidants, ‘Bingli’ was considered a suitable representative cultivar for further qualitative profiling. This selection does not imply that ‘Bingli’ was highest for every parameter, nor that the LC-MS profile represents all eight cultivars.

Preliminary qualitative screening via UHPLC-Q-TOF/MS provided a tentative profile of the phenolics in the ‘Bingli’ cultivar. The potential presence of several hydroxycinnamic acid derivatives, including isomers of p-coumaroylquinic acid and 1-caffeoyl-4-deoxyquinic acid, along with A-type procyanidin dimers (*m*/*z* 575), trimers (*m*/*z* 863), and tetramers (*m*/*z* 1151), reflects the chemical complexity of both litchi pericarp and seeds. Although these compounds were tentatively identified based on mass-to-charge (*m*/*z*) ratios and fragment information and their structural assignments have not been confirmed with authentic standards, their detection is consistent with the high TPC observed across the tissues. Confirmation with authentic standards is needed in future work, particularly for isomeric compounds.

Consistent with these structural indications and previous reports [10,21,34,35,36], targeted HPLC analysis used Procyanidin A2 and B2 as primary biomarkers. The quantitative results showed that Procyanidin A2 concentration varied across litchi tissues, as seeds contained a higher concentration than pericarps. Furthermore, the concentration differences among litchi cultivars were significant. However, Procyanidin B2 was not detected in any of the samples under the current analytical conditions, which may indicate that its concentration was below the detection capability of the analytical procedure used, although the limit of detection was not formally determined in this study. The result is consistent with findings obtained by Li et al. [21], who reported that Procyanidin B2 contents were significantly less abundant than Procyanidin A2 in litchi pericarp. To enhance analytical sensitivity for compounds such as Procyanidin B2, condensation treatment such as vacuum distillation or solid-phase extraction can be applied in the future.

### 4.4. Implications for Food Industry and Environmental Sustainability

The antioxidant capacity of litchi fruit by-products suggests their potential as candidate natural food additives [37]. As indicated in the introduction, the food industry requires safe alternatives to synthetic antioxidants (e.g., BHT) to inhibit ROS-induced lipid oxidation. The free-radical scavenging activities observed in the extracts suggest that they may have potential to inhibit lipid oxidation in lipid-rich foods. If confirmed in real food systems, this could help address safety concerns associated with synthetic additives [13,38]. However, this possibility remains hypothetical and requires verification in real food model systems, since activity in FRAP, ABTS, and DPPH assays does not directly demonstrate efficacy in preventing rancidity.

The differential phytochemical distributions between the pericarp and seeds may provide different technological functions. Litchi pericarp is rich in phenolic compounds and could potentially serve as a natural colorant or antioxidant ingredient, replacing synthetic dyes while providing supplementary antioxidant benefits to food formulations [39]. Meanwhile, due to their high levels of oligomeric procyanidins, litchi seeds may be considered candidate raw materials for future nutraceutical research. However, their efficacy, safety, bioaccessibility, and activity in biological systems require further evaluation before nutraceutical applications can be confirmed [12].

Furthermore, from an environmental perspective, the application of litchi fruit by-products reduces the environmental impact of their disposal. Currently, the extensive volume of discarded pericarp and seeds contributes to greenhouse gas emissions when managed through landfilling [40]. Upcycling these agro-industrial residues into functional food ingredients represents a practical application of the circular economy. This strategy reduces the environmental footprint of fruit processing and supports broader sustainable food system goals [3,41,42].

However, polyphenols can degrade easily during thermal processing, light exposure, and gastrointestinal digestion [43]. This instability can limit their commercial applications and reduce their bioaccessibility. Future studies should focus on encapsulation technologies, such as microencapsulation or nanoemulsions, to preserve the structural integrity and antioxidant efficacy of litchi-derived polyphenols within food matrices [44].

### 4.5. Limitations

Several limitations should be considered when interpreting the results. Each cultivar was represented by one commercial market batch obtained on one of two purchase dates. Cultivar effects therefore cannot be separated fully from batch, harvest maturity, cultivation conditions, or postharvest history. Maturity was assessed visually from pericarp color because objective indices and exact harvest information were unavailable. The findings should thus be viewed as comparisons among the commercial samples tested rather than as a controlled varietal experiment.

Sample preparation and analytical methods may also have influenced the findings. Hot-air drying at 60 °C may have degraded or transformed heat-sensitive phenolics. Because residual moisture was not measured and no fresh or freeze-dried control was included, the magnitude and direction of this effect cannot be determined. Only one extraction protocol was used and extraction recovery was not assessed. The Folin–Ciocalteu and chemical antioxidant assays are method-dependent, while the Pearson correlations, based on eight cultivars per tissue, indicate association rather than causation. Accordingly, the reported values apply to the oven-dried samples under the stated conditions, and cross-study comparisons should be made cautiously.

The chemical characterization and proposed applications require further validation. LC-MS profiling was limited to ‘Bingli’, and most compounds were tentatively annotated without authentic standards; therefore, this profile cannot be assumed to represent all cultivars. The proposed food, nutraceutical, and environmental applications were not tested in food models, biological systems, safety studies, or process-level environmental assessments. Future studies should include independently replicated batches, objective maturity and moisture measurements, appropriate drying controls, broader compound confirmation, and application-based validation.

## 5. Conclusions

This study evaluated the in vitro antioxidant capacities and phenolic profiles of pericarp and seed extracts from eight commercial litchi cultivars, with significant variations observed among cultivars and between tissue types. The ‘Bingli’ and ‘Xianjinfeng’ cultivars exhibited relatively high TPC and antioxidant activity. Among the targeted biomarkers, Procyanidin A2 was quantified in all tested samples, especially in litchi seeds compared with litchi pericarp. Overall, these findings suggest that litchi fruit by-products, particularly the seeds of ‘Bingli’ and ‘Xianjinfeng’, are rich in phenolic compounds and exhibit good in vitro antioxidant capacity. Therefore, they may be considered as candidate natural antioxidant sources for further investigation. However, the present results are limited to in vitro assays, and studies using food model systems, bioaccessibility assessments, and biological validation are required before any practical food or nutraceutical application can be proposed.

## Figures and Tables

**Table 1:** Tentatively annotated phenolics in the pericarp and seeds of ‘Bingli’ based on qualitative LC-MS screening.

Name	Formula	*m*/*z*	Pericarp/Seeds
*p*-Coumaroylquinic acid isomer(s)	C_16_H_18_O_8_	337.0929	Pericarp and seeds
1-Caffeoyl-4-deoxyquinic acid	C_16_H_18_O_8_	337.0929	Pericarp and seeds
Procyanidin A-type dimer(s)	C_30_H_24_O_12_	575.1190	Pericarp and seeds
Procyanidin A-type trimer(s)	C_45_H_36_O_18_	863.1806	Pericarp and seeds
Procyanidin A-type tetramer(s)	C_60_H_48_O_24_	1151.2414	Pericarp

Note: Compounds were tentatively annotated based on accurate mass and fragment information matched with databases (METLIN, ChemSpider, HMDB) and literature data. Authentic standards were not available for most compounds; therefore, structural assignments and positional isomers could not be confirmed. Entries such as *p*-coumaroylquinic acid isomer(s) or procyanidin A-type dimer(s) indicate that multiple isomeric forms may be present but were not differentiated under the current analytical conditions.

## Data Availability

The data presented in this study are available on request from the corresponding author.

## Supplementary Materials

The following supporting information can be downloaded at: https://www.mdpi.com/article/10.3390/antiox15091187/s1, Table S1: Tentative phenolic profiling results of the ‘Bingli’ cultivar by-products based on LC-MS.
