# Supplementary material for: Discovery and Characterization of Antioxidant Compounds in the Pericarp and Seed Byproducts of Different Litchi (Litchi chinensis Sonn.) Fruits
Source: Antioxidants (Basel). 2026 Sep 17;15(9):1187. doi: 10.3390/antiox15091187 (PMC13603736; doi:10.3390/antiox15091187)
Supplement: Supplementary file 1 [file antioxidants-15-01187-s001.zip › antioxidants-4516930-supplementary.pdf]

Supplementary Materials

Table S1. Tentative phenolic profiling results of the 'Bingli' cultivar by-products based on LC-MS.

Name	Formula	*m/z*	Tissue detected
4-Hydroxycatechol	$C_6H_6O_3$	171.0297	Pericarp
Pyrogallol	$C_6H_6O_3$	171.0297	Pericarp
Phloroglucinol	$C_6H_6O_3$	171.0297	Pericarp
(Z)-Tamarindienal	$C_6H_6O_3$	171.0297	Pericarp
Cinnamoylglycine	$C_{11}H_{11}NO_3$	250.0720	Pericarp
N-feruloylglycine	$C_{11}H_{11}NO_3$	250.0720	Pericarp
4-Hydroxy-3-methoxy-cinnamoylglycine	$C_{12}H_{13}NO_5$	250.0720	Pericarp
Bergenin	$C_{14}H_{16}O_9$	327.0719	Seeds
Vanillin glucuronide	$C_{14}H_{16}O_9$	327.0719	Seeds
(S)-Angelicain	$C_{15}H_{16}O_6$	337.0929	Both
trans-Grandmarin	$C_{15}H_{16}O_6$	337.0929	Both
1-Caffeoyl-4-deoxyquinic acid	$C_{16}H_{18}O_8$	337.0929	Both
trans-5-O-(4-coumaroyl)-D-quinic acid	$C_{16}H_{18}O_8$	337.0929	Both
3-O-*p*-coumaroylquinic acid	$C_{16}H_{18}O_8$	337.0929	Both
5-O-*p*-coumaroylquinic acid	$C_{16}H_{18}O_8$	337.0929	Both
4-O-*p*-coumaroylquinic acid	$C_{16}H_{18}O_8$	337.0929	Both
Hydrojuglone glucoside	$C_{16}H_{18}O_8$	337.0929	Both
Procyanidin A2	$C_{30}H_{24}O_{12}$	575.1190	Both
Epicatechin-(2beta->7,4beta->6)-catechin	$C_{30}H_{24}O_{12}$	575.1190	Both
Procyanidin A1	$C_{30}H_{24}O_{12}$	575.1190	Both
Procyanidin A5'	$C_{30}H_{24}O_{12}$	575.1190	Both
Pavetannin A2	$C_{30}H_{24}O_{12}$	575.1190	Both
Pavetannin B6	$C_{45}H_{36}O_{18}$	863.1806	Both
Cinnamtannin B1	$C_{45}H_{36}O_{18}$	863.1806	Both
Cinnamtannin D1	$C_{45}H_{36}O_{18}$	863.1806	Both
Pavetannin B2	$C_{45}H_{36}O_{18}$	863.1806	Both
Cinnamtannin B2	$C_{60}H_{48}O_{24}$	1151.2414	Pericarp
Cinnamtannin D2	$C_{60}H_{48}O_{24}$	1151.2414	Pericarp
Pavetannin C1	$C_{60}H_{48}O_{24}$	1151.2414	Pericarp
